# Reflective practice and transcultural psychiatry peer e-learning between Somaliland and the UK: a qualitative evaluation

**DOI:** 10.1186/s12909-020-02465-y

**Published:** 2021-01-15

**Authors:** Mia Prosser, Thomas Stephenson, Jai Mathur, Hanieh Enayati, Abdirasak Kadie, Manal Mohamed Abdi, Jibril I. M. Handuleh, Roxanne C. Keynejad

**Affiliations:** 1 King’s College London, London, UK; 2 South London and Maudsley NHS Foundation Trust Maudsley Hospital, Denmark Hill, London, Greater London SE5 8AZ UK; 3 St George’s, University of London, London, UK; 4 Buhoodle District Hospital, IOM Mida Finnsom Health Project, Bohotle, Somaliland; 5 College of Medicine and Health Science, University of Hargeisa, Hargeisa, Somalia; 6 College of Health Sciences, Amoud University, Borama, Somaliland; 7 Department of Psychiatry, St Paul’s Hospital Millennium Medical College, Addis Ababa, Ethiopia; 8 Section of Women’s Mental Health, Health Service and Population Research Department, Institute of Psychiatry, Psychology & Neuroscience, King’s College London, De Crespigny Park, London, SE5 8AF UK

**Keywords:** Somaliland, Medical education, Reflective practice, Peer-to-peer education, E-learning, Transcultural psychiatry, United Kingdom, Health partnerships, Intercultural awareness, Intercultural practice

## Abstract

**Background:**

Reflective practice is a key skill for healthcare professionals. E-learning programmes have the potential to develop reflective practice in remote settings and low- and middle-income countries (LMICs), where access to in-person reflective groups may be reduced. ‘Aqoon’ is a global mental health peer-to-peer e-learning programme between Somaliland and UK medical students. We aimed to explore participants’ experiences of participating in the Aqoon programme, including their experiences of reflective practice.

**Methods:**

Thirty-three medical students (22 Somaliland, 11 UK) enrolled in Aqoon. We matched volunteer learners in trios, to meet online to discuss anonymised clinical cases relevant to chapters of the World Health Organization’s mental health gap action programme (mhGAP) intervention guide. We conducted thematic analysis of learners’ reflective writing and post-programme focus group transcripts.

**Results:**

Twenty-four students (73%) attended at least three online discussions (14 Somaliland, 10 UK). Somaliland and UK students described improved reflective skills and greater recognition of stigma towards mental ill-health. Themes included gaining memorable insights from peer discussions which would impact their medical education. UK students emphasised improved cultural understanding of common psychiatric presentations whilst Somaliland students reflected on increased clinical confidence.

**Discussion:**

Integrating reflective practice into Aqoon showed the potential for low-cost e-learning interventions to develop cross-cultural reflective practice among medical students in diverse settings.

## Background

### Somaliland

Somaliland is a self-declared independent region in Somalia with an estimated population of 2–3.5 million [1]. The trained public sector psychiatric workforce is small, leaving a large unmet need for mental health care [2]. A situational analysis of mental health services in Somalia highlighted a lack of culturally-adequate treatments and community-based services integrated into primary care [3]. To date, reflective practice is not utilised within medical curricula in the region. King’s Somaliland Partnership (KSP) is a long-term clinical education collaboration between hospitals and universities in Somaliland and King’s Health Partners (UK), which aims to improve healthcare outcomes by strengthening people, organisations and systems [4, 5].

### KSP

KSP is a global health partnership department at King’s College London department of global and population health.

### Aqoon

‘Aqoon’, meaning *knowledge* in Somali, is a peer-to-peer global mental health e-learning programme established by KSP in 2010 [6]. The programme matches medical students at four universities across Somaliland and UK for weekly small-group, online case-based discussion of clinical cases in psychiatry, addressing topics selected from the World Health Organisation (WHO)‘s mental health gap action programme (mhGAP) intervention guide [7, 8].

### Reflective practice

Learning to reflect enables healthcare professionals to understand the meaning of complex situations and facilitates deeper learning from experience [9]. Reflective practice is an important skill for all clinicians [10], although most evidence for its role in undergraduate medical education comes from high-income country (HIC) settings rather than low- and middle-income countries (LMICs) [11]. Reflective practice-based approaches to medical undergraduate training have recently been reported in diverse settings, from Taiwan [12, 13] to Thailand [14] and South Africa [15]. One American study proposed a tool to measure reflective capacity among medical students [16]. However, the objective assessment of reflective practice skills in this population is confounded by the subjective nature of reflective content [17]. We therefore explored students’ subjective experiences of learning reflective practice skills through Aqoon.

### Intercultural E-learning

Expanding access to mobile technology has coincided with growing interest in web-based medical education and changes to the types of resources medical students use to learn new knowledge [18]. One UK study found that reflective practice could be developed by asking students to submit reflections on their learning by email [19]. Applications of e-learning in LMIC settings [20] and partnerships between HICs and LMICs [21] suggest that online education can be feasible in resource-limited settings despite internet bandwidth and connectivity difficulties.

Intercultural skills are crucial for healthcare professionals, who work with patients and colleagues from diverse cultural backgrounds [22, 23]. Definitions of intercultural competence vary, although one Delphi study of intercultural administrators and academics identified common themes of “awareness, valuing, and understanding of cultural differences; experiencing other cultures; and self-awareness of one’s own culture” [24]. E-learning interventions between HICs and LMICs can facilitate experience of other cultures and improve understanding [25]. For example, one study of Australian and Indonesian medical students found that an online group global health intervention helped learners to critically engage with different cultural approaches to global health topics [26].

There is some evidence that intercultural e-learning during psychiatric undergraduate education can foster student reflection and improve intercultural awareness [27]. Training clinicians was identified as one of five priorities for global mental health by a Lancet Commission, which highlighted technological innovation as an important solution [28]. Innovative approaches to mental health education are particularly important in LMIC settings, where numbers of practising psychiatrists can be extremely low [29]. A systematic review of interventions to engage students with psychiatry recommended implementing novel programmes alongside in-depth clinical placements and enrichment activities to improve attitudes towards the specialty [30]. Aqoon therefore additionally aimed to engage Somaliland and UK medical students with the relevance of psychiatry to wider medical practice.

## Methods

### Context

We used the low-bandwidth ‘Medicine Africa’ website to facilitate e-learning between medical student peers in Somaliland and the UK [31]. The website had been redeveloped, offering a choice between audio/video-assisted communication and free-text discussions, when internet connectivity is limited.

### Aim

We aimed to explore the experiences of students participating in the Aqoon programme, including their experiences of reflective practice.

### Recruitment

We recruited volunteer participants from four medical schools: Hargeisa and Amoud Universities in Somaliland and King’s College London (KCL) and St George’s University of London (SGUL) in the UK. We advertised Aqoon using internal email, lecture announcements and social media posts. Students applied by submitting a paragraph outlining their motivation to participate and were selected by a UK course leader (TS). We randomly assigned students to groups of three for the duration of the programme in a ratio of two Somaliland to one UK student. Thirty-nine students applied to take part (28 Somaliland, 11 UK) and 33 (22 Somaliland, 11 UK) were accepted. Six applicants were rejected because their statement of interest showed a misunderstanding of the aims of the programme. Applicants needed to be in at least their second year of training to be eligible, although all applications received from Somaliland were from fifth- and sixth-year students. More Somaliland participants were male whilst more from UK were female (see Table 1).

**Table 1:** Aqoon participant demographic information

	Somaliland	UK	Total
All participants	22	11	33
Gender	M 19; F 3	M 3; F8	M 22; F11
Year of training (6th = final year)	Y2: 0	Y2: 1	Y2: 1
	Y3: 0	Y3: 7	Y3:7
	Y4: 0	Y4: 2	Y4: 2
	Y5: 13	Y5: 1	Y5: 14
	Y6: 9	Y6: 0	Y6: 9
Participants completing programme	14	10	24
Gender	M 13; F1	M3; F7	M16; F8
Year of training (6th = final year)	Y2: 0	Y2: 1	Y2: 1
	Y3: 0	Y3: 6	Y3: 6
	Y4: 0	Y4: 2	Y4: 2
	Y5: 7	Y5: 1	Y5: 8
	Y6: 7	Y6: 0	Y6: 7

### Learning objectives and session content

Participants were informed that Aqoon’s learning objectives were to:

Cluster 1 – Clinical:

1. Improve clinical reasoning skills, applied to peer-led clinical case-based discussions.
2. Increase confidence in clinical contact with people presenting with mental health problems.
3. Increase familiarity with common mental health presentations to emergency and primary care settings in particular where cultural differences exists between patients and mental health workers.
4. Provide learners with knowledge and experience of the WHO mhGAP intervention guide (mhGAP-IG) and its application by front-line clinicians in LMICs.

Cluster 2 – Transcultural:

5. Enhance understanding of cross-cultural differences in mental health service provision in Somaliland and the UK.
6. Share knowledge of global differences in how mental health problems are culturally conceptualised.
7. Foster reflection on stigma and discrimination towards people with mental health problems, through open dialogue with trusted peers.

Cluster 3 – Collaborative learning:

8. Develop a collaborative educational relationship with a peer overseas.
9. Contextualise medical education within a global profession, through an environment of mutual respect and collaboration.

Session content comprised a new mental health topic every week, selected from the WHO mhGAP intervention guide 2.0 (mhGAP-IG) [32]. The mhGAP-IG curriculum addresses the assessment and integrated management of priority mental, neurological and substance use disorders in LMICs and provides clear protocols for clinical decision making for non-specialist primary care workers [7]. Following discussion between Somaliland and UK organisers, including TS who had prior experience of both participating in and organising Aqoon, the mhGAP chapters selected for Aqoon sessions were (i) psychosis, (ii) depression, (iii) substance use disorders, (iv) child and adolescent mental health, (v) old age psychiatry, (vi) eating disorders, and (vii) anxiety disorders including post-traumatic stress disorder. Each session began with a fictitious case-based discussion, structured around clinical questions about diagnosis and management and reflective prompts inviting discussion of cultural factors influencing each topic. We provided trios with two resources: a session guide containing a case study and question prompts for that week’s topic (see supplementary document), and the WHO mhGAP-IG, around which the course curriculum was structured [32]. Somaliland course facilitators fed back on content and presentation of session guides leading to minor amendments before the programme started.

### Course structure

We instructed student trios to meet online for seven weekly one-hour online sessions using the low-bandwidth website, MedicineAfrica.com [31]. Trios met online at pre-arranged times and communicated via live video-call or live chat functions, depending on internet quality, using tutorial resources to guide their discussions. No teaching staff facilitated sessions, but students were able to contact the coordinating psychiatrists (TS and JH) via the organising representatives, if needed. Students were informed in advance of the requirement to submit a piece of reflective writing to complete the programme. They were also required to complete a before-and-after evaluation questionnaire. For many participants, particularly from Somaliland, this was their first experience of reflective writing. Therefore, we offered an optional teaching session introducing reflective practice concepts to the Somaliland group. This session was developed and delivered by the Somaliland supervisor (JH) who was not involved in the analysis and was attended by five students.

### Evaluation

Building on previous mixed methods evaluations of Aqoon [27, 33], we used qualitative analysis of reflective writing by participants and transcripts from focus groups to explore students’ experiences of cross-cultural learning, allowing participants to express diverse and unforeseen perspectives [34]. Qualitative methods are best suited to understanding the impact of novel interventions like Aqoon, incorporating reflective practice elements for the first time. We approached the analysis from an interpretivist paradigm in which we were interested in the experiences of reality constructed by learners in diverse cultural and educational contexts. We considered source triangulation particularly important because group discussions could remind students of points they might have forgotten to discuss in their written reflections.

We therefore evaluated Aqoon using thematic content analysis of data from two sources. First, learners submitted a 400-word piece of reflective writing about their experience of Aqoon. They were encouraged to reflect on:

- What stood out in their discussions of cultural differences?
- Their experience of identifying and resolving differing cultural perspectives on clinical questions with their partner.
- Whether sessions were productive and when they were unproductive.
- Whether sessions had an impact on their medical education.
- Whether sessions had an impact on their reflective capacity.

Second, a sub-group of volunteer participants attended one of two Medicine-Africa based online focus groups (a typed live-chat discussion attended by Somaliland students and an audio conference call attended by UK students, based on internet bandwidth). We allocated learners to trios at random (with a balance of Somaliland and UK learners) and allocated a focus group facilitator to each (MD for Somaliland and MP for UK students). Focus group size was informed by recommendations that four to eight participants is optimal [35]. Facilitators received online focus group training from the Somaliland supervisor (JH) and followed the same topic guide. The topic guide focused on two discussion topics: first, the experience of taking part in Aqoon and second, and its effects on learners’ cultural conceptualisations of mental health. Participants were asked for examples of their discussions on intercultural issues, differences in attitudes from their partners, the effect of those discussions on their own perspectives, whether they impacted on their wider medical education and any lessons learned.

Following consultation with the KCL Psychiatry, Nursing and Midwifery Research Ethics Subcommittee (PNM REC) and Amoud University in Somaliland, this evaluation was assessed as an educational service evaluation, for which ethical approval was not required.

### Data collection and analysis

With students’ agreement, we collated anonymous written reflective pieces alongside transcripts of the Somaliland student and (audio-recorded) UK student focus groups. One participant’s written reflection was omitted from the analysis as they did not consent. Analysts included Somaliland (AK, MD) and UK authors (MP, TS), who performed thematic analysis using computer-assisted qualitative analysis software (NVivo) in Somaliland and UK sub-groups. We applied investigator triangulation, with AK and MD analysing data independently to MP prior to discussion, to increase sensitivity to culture-specific information and meaning within the data. Following discussion between analysts, we integrated and cross-referenced emergent codes, to identify over-arching themes. MP and TS subsequently triangulated findings with qualitative data from the two focus groups.

## Results

Twenty-four of 33 students (73%) attended at least three online discussions (14 Somaliland, 10 UK), of whom 19 (79%) completed all evaluation requirements including a written reflective account. Five Somaliland students attended the typed live-chat focus group discussion and four UK students attended the audio-facilitated online focus group.

### Written reflections

Key themes that emerged during analysis of students’ reflective accounts included cultural exchange, productivity during sessions, developing reflective practice and the impact of Aqoon on wider medical education.

#### Cultural exchange

Students described an enjoyable cultural exchange, which enabled them to share their personal perspectives and gain insights into how their own culture responds to people with mental health problems. Somaliland students highlighted that ‘cultural interventions’ were commoner responses to mental ill-health than biomedical treatments. For example:

*In UK where my colleague lives [they] told me that there are no cultural interventions for one’s diagnosis and [it] is managed according to his condition and he can get a medical help from the nearest hospital, that was what stood out on me during our discussion … we discussed about culture but in perspective of how culture affects patients’ condition and treatment.*

Somaliland medical student

One Somaliland student described these discussions as like ‘holding up a mirror’ to see what ‘we can learn’ from their peers, although many learners described a two-way learning process. UK students tended to reflect on how their approach to cases was influenced by their own background. Students from both countries reflected on differences in diagnostic and treatment models commonly used by their respective mental health services, comparing a focused biomedical approach in Somaliland to a more bio-psycho-social model in the UK. One student used the example of someone experiencing a panic attack:

*This opportunity has allowed me to broaden my understanding of both the cultural factors influencing the presentation of mental illness, and a community’s response to it. In particular, I found it very interesting to discuss our different personal experiences witnessing an individual experiencing a panic attack.*

UK medical student

Students from both countries valued transcultural exchanges with their peers. Some Somaliland students expressed a wish to embrace the UK model, to better understand patients presenting with mental health crises. A common theme among learners from both countries was the intention to apply something learned from their overseas peer or reflected upon afterwards, into their future practice. UK students enjoyed sharing clinical knowledge and experiences with their more senior counterparts from Somaliland.

Students singled out their case discussions on anorexia nervosa (*n*=6), substance misuse disorders (n=6) and dementia (*n*=5) as particularly memorable. Nine students highlighted their discussions around service provision as positive. Some UK students were previously unaware that eating disorders were more common in high-income countries than LMICs, and reflected on possible reasons. Learners noted differing views to their peers about substance use disorders, especially regarding the extent of personal responsibility for addictions. Many students described memorable discussions on psychiatry in older adults, particularly regarding cultural attitudes to caring for elderly relatives and the impact on service provision. Somaliland students expressed surprise at older people living in care homes rather than with their families; some expressed pride in their nation’s approach. Similarly, UK students often reflected on negative implications of professional care for elderly people, such as depersonalised care compared to that provided by families.

Discrimination and stigma towards mental health disorders were explicitly raised by half of respondents (*n*=12). UK students reflected on mental health stigma at home while acknowledging the attention paid to patients’ human rights. One UK student reflected that ‘stigma is certainly not a “western phenomenon” and seems to transcend cultural barriers’. Somaliland students reflected on the extent to which society reinforces misconceptions of people with mental ill-health, blaming the media, government, religious institutions and lack of education.

Learners expressed pleasure in finding many things in common with their overseas peers. Whilst some UK students struggled with differing views on religious perspectives on health, Somaliland students did not raise this issue. However, respondents generally considered differences to enhance their learning experience:

*We discussed sexual relationships which was delicate because I didn’t want to offend my partner with my views. In the end, he wasn’t offended at all which gave me more confidence to state how I feel in future sessions.*

UK medical student

*We were thinking, things will be different about the subject since we are in different schools and different continents. However, we realized we even had same terminology and same approach.*

Somaliland medical student

#### Productivity of sessions

Respondents varied in the style of questions they preferred for discussion prompts, between focused clinical questions and open, reflective questions. Some learners thought that structured questions (asking about treatment options or how to elicit symptoms) helped to focus discussions. Most favoured open questions (asking them to reflect on and share experiences from their respective cultures) for prompting richer interactions.

Some students would have valued more guidance on how to approach peer discussions and written guidance on their partner missing a session. Others identified logistical issues such as internet connectivity problems as a barrier to meaningful discussion.

#### Positive impacts

Most respondents described enjoying Aqoon, without direct prompting. Themes included enjoying learning from peers of a different cultural background and gaining skills relevant to clinical practice. No student reported a negative experience.

*Overall, the Aqoon programme has been an utter privilege to be involved with and I would recommend it highly to any curious medical student.*

UK medical student

When asked about Aqoon’s impact on medical education, many students responded positively and none negatively. Somaliland respondents reported improving their psychiatric clinical reasoning skills, developing their confidence in approaching patients, and improving their clinical knowledge. UK students recalled that the programme developed non-clinical skills and knowledge such as interpreting patient experience with greater empathy and broadening the context in which they can understand patients from diverse backgrounds.

*The experience made me aware of the expectations that different cultures have of medical professionals, and how I might incorporate that in future practice. Minority groups are overrepresented in mental health services, so this makes an appreciation for cultural differences in the presentation of mental illness especially important.*

UK medical student

#### Reflective practice

Most learners described improving their reflective practice skills, irrespective of their prior experience. Somaliland students expressed the novelty of engaging in reflective practice, mostly for the first time. UK students, all of whom had at least one prior experience of formal reflection, also described increased reflective capacity during Aqoon:

*There was a significant difference between our first and last session. The first sessions we spent a lot of time breaking down how to take the history and manage the patient’s condition. In later sessions we took a more reflective approach; we discussed more around the differences in our medical school educations and thoughts around cultural influences.*

UK medical student

### Focus groups

Themes that emerged during focus group discussions triangulated with perspectives expressed in students’ reflective accounts: the value of cultural exchange and intercultural learning, discussing specific topics cross-culturally, and the overall positive experience. As in reflective accounts, learners highlighted wider benefits to their medical education but Somaliland students emphasised increased clinical confidence whilst UK students valued benefits to their future careers.

### Somaliland students

Students highlighted the course structure and use of reflective practice as positive aspects of their Aqoon experience, during focus groups:

*[We had the chance to] study Kolb’s theory and reflective writing.*

*The method of studying was different. Peer group was good. And partner learning together has increased our experience toward the psychiatry.*

Students highlighted transcultural learning:

*[I learned] not just about the psychiatry but also cultural and general knowledge… and learning the difference and respecting it.*

*Here mental illness is sometimes interpreted as jinn [demonic] possession.*

Stigma and shame, although not mentioned in many Somaliland students’ written reflections, was raised in the focus group:

*Stigma is high in our setting... Less... In others…Yes, one benefit [of Aqoon is] to promote in our area to stop stigma.*

The Somaliland focus group praised the same weekly topics as written reflections: old age psychiatry, eating disorders and substance use disorders:

*Alcohol consumption was rare to us and my partner taught me well while I did [taught them about] Khat abuse.*

*Major problems of England were eating disorders which is rare for us.*

*On the other hand, dementia is more common in UK and they have places to care [for] these patients, unlike Somaliland where these patients are kept in homes.*

One student described learning to separate their personal beliefs from clinical interactions:

*Yes, the alcohol abuse... It had effect on me... How can I treat a patient who is drunk... When I [am] still thinking he is evil…So I think, when my partner had said a lot about the culture there and how alcohol is common it changed me because I shouldn’t think as a religious [person] but as a doctor and professionally I should treat [them] as other cases.*

Students discussed how their clinical decision making and management skills developed over the course:

*If I had met a case who is planning suicide, I previously let him/her to go… but now [I would] admit [them to hospital].*

*We get some experience [of] how to manage and deal with [a] patient having or suffering mental health disorder.*

Students reported increased clinical confidence when encountering people with mental health problems:

*I still had lots [of] fear... There was a guy who had hit me with a large stick... From that [time] on … I don’t trust them... So yes, now I can meet with them [people with mental health problems]… Good change.*

*[I gained] learning more about psychiatry... and feeling more confident in terms of patient approach actually.*

#### UK students

The UK focus group raised common themes with the Somaliland discussion, with greater emphasis on cultural exchange:

*My favourite bit was when we were teaching each other about our different cultures, and they taught me some words.*

*I liked seeing the things we did differently/perceived differently and the stuff that was the same also … I realised how easy it was to chat to them and had loads in common I wasn't expecting.*

As described in reflective accounts, focus group participants agreed that religion was a source of discordant views:

*They were saying in their culture, people who are mentally ill are often badly religious people, so it is viewed almost as a punishment from Allah, so it was just interesting to see their viewpoint of it.*

The UK focus group highlighted the same weekly topics as Somaliland students:

*[In] the eating disorders week we had a really thoughtful discussion, and it was really interesting: they were saying they just don’t have it in their country at all, it just felt like a very foreign concept, and that week coincidentally I had a lecture in my psychiatry teaching about eating disorders and the high prevalence of BME [black and minority ethnic people with] eating disorders in South London, and I was saying this and they were really surprised and it really challenged their perceptions of what eating disorders might be, it just felt quite informative and people were getting engaged so that was good.*

*We discussed the care of the elderly, I think it was when we were doing dementia perhaps, I don’t know the specific topic but we definitely got on to that, and they were saying how, you know there is no specialist dementia care home and they’re not in hospital, they’re just looked after at home by the families, and the kind of strain that puts on the family dynamic.*

*I remember that actually, they talked about [how] the children usually fight to be the one to be able to care for the parents.*

*I really liked the week we talked about substance abuse, because it was really interesting to see how it was totally different substances obviously in the two countries, and I guess the cultural reactions to it, like what we thought of it was very different.*

UK respondents expanded on Aqoon’s impact on their future careers, raised in their written reflections:

*For me the program sort of opened up my mind a bit more to thinking about helping other countries with the lack of mental health facilities that they have and more of a biological understanding rather than a psychosocial understanding. I think I’ve become more culturally aware especially to people from Somalia and more patient, if you like. Things like that are going to happen in clinical practice, maybe if you’re having an issue with Language Line [telephone translation] or if a concept isn’t understood by a patient or you, it will take more time. I definitely think it’s affected me in that way.*

## Discussion

This transcultural peer-to-peer e-learning partnership was enjoyed by Somaliland and UK students alike. Both groups of learners, including Somaliland students for whom reflective writing was novel, and UK students with more experience thereof, reported that Aqoon helped to develop their reflective skills. Both groups felt they had gained something from the programme, with Somaliland students tending to focus on improvements in clinical confidence and decision-making whilst UK students emphasised increased cultural awareness and clinical knowledge. Differences in the stages of training of Somaliland and UK learners might have contributed to these divergences, with Somaliland students generally closer to completing their undergraduate medical training and more focused on the importance of clinical decision-making than UK students.

Mutual recognition of mental health stigma in both cultures enabled students to reflect on commonalities as well as differences. Some UK students expressed surprise about their shared attitudes and the ease of communication, suggesting that Aqoon challenged participants’ preconceptions about their partners’ cultural beliefs and the extent of transcultural differences. Discussions – and, occasionally, disagreements – about issues surrounding dementia care, substance use and eating disorders in particular enabled learners to reflect on cultural conceptualisations of mental health problems. Themes emerging from written reflections were supported by triangulation with focus group discussions. Written session guides using simulated case studies with diagnostic, management and reflective prompts facilitated problem-based peer learning. These findings are consistent with a previous evaluation of Aqoon (matching students in pairs), demonstrating that this peer learning model was effective using trios [27]. Some students preferred free-flowing discussion to structured prompts. Learners from both countries accepted the curriculum structured around the WHO mhGAP-IG; to our knowledge, Aqoon is the first intervention to use e-learning to sensitise medical students to this eminent clinical tool [7]. Students discussed sensitive subjects such as religious beliefs professionally, with shared, respectful curiosity.

Aqoon may have translational benefits for the global mental health education of trainee healthcare professionals. Increased clinical confidence, enhanced reflective skills and ability to challenge one’s own preconceptions, all benefit professional practice. Aqoon’s use of course facilitators but not tutors, a freely available low-bandwidth e-learning platform and open-source WHO curriculum makes it cheap to deliver and sustainable to implement. Aqoon’s simulated, practical use of the mhGAP-IG may increase its uptake among course alumni, most of whom will practice medicine outside of specialist psychiatric services. The Aqoon model is also notable for being a deep collaboration between its Somaliland and UK partners with shared decision-making across its design, operation and evaluation.

### Implications

Our evaluation of Aqoon suggests several notable features valued by learners. Students valued the small group learning format, enabling trios to become personally acquainted and contribute to open discussion among trusted peers. Despite MedicineAfrica’s low bandwidth design, internet access remains a logistical barrier to implementation. Matching peer learners in trios largely overcame challenges from previous years of sessions not taking place when one student was unable to log on, and learners reported logistical difficulties with meeting less frequently under this format than in the past [6]. Aqoon benefitted from well-established Somaliland-UK links via King’s Somaliland Partnership (KSP), enabling leadership and recruitment to be organised and supported by existing KSP volunteers. The session guide, co-authored by Somaliland and UK organisers with experience of clinical psychiatry, ensured that peers followed a standardised curriculum. Balancing structured clinical questions to develop knowledge and reflective prompts to foster discussion ensured relevance of learning to practice. Previous iterations of Aqoon have recommended up to ten online sessions. The goal of seven sessions in this course was more achievable for participants, with lower drop-out rates than previous years.

### Strengths and limitations

Investigator triangulation between Somaliland and UK analysts, and source triangulation using written reflections and focus group transcripts both enhance the validity of our findings, allowing greater depth and breadth of participant feedback. Somaliland and UK analysts maintained reflexivity by considering their own perspectives as undergraduate students or trainee psychiatrists based in Somaliland and the UK [36]. Once analysts’ lists of codes had been cross-referenced, few additional themes arose, suggesting that data saturation was achieved. Longer reflective accounts might have enhanced saturation but was balanced against reasonable expectations of students’ time commitments. Aqoon was run entirely in English: a second language for Somaliland students. Conducting reflective writing and focus groups in participants’ first language may have increased the richness and depth of reflective accounts, but resources for appropriate translation were not available.

One simulated case (anorexia nervosa) was intentionally selected to facilitate discussion about the impact of culture on mental health in countries like the UK. The case, focused on a problem rarely encountered in Somaliland, received positive feedback from Somaliland students; the choice of cases and presentation of course materials was positively evaluated by learners.

Both focus groups took place via MedicineAfrica.com, using typed live chat for Somaliland and audio recording for UK students. The use of computer-mediated focus groups has been empirically evaluated and deemed a convenient alternative to in-person meetings [37, 38], including live chat formats [39]. However, the difference in methods used between the two groups may have influenced the content of discussions. This study did not explore the experiences of the 27% of participants who dropped out of the programme, who may have had a different experience.

## Conclusions

The organisation, implementation and evaluation of the Aqoon peer-to-peer global mental health e-learning programme is a collaborative endeavour, which demonstrated the relevance of reflective practice to intercultural e-learning. Engaging trainee healthcare professionals with mental health in a global context is a key priority [28]. Students participating in Aqoon reported improved reflective skills. Our findings suggest that important transcultural clinical skills could be fostered across global healthcare, through low-resource, online, peer-directed educational partnership. This hypothesis requires further research investigation, with larger sample sizes. Unanswered questions include what long-term impact transcultural and reflective skills derived from online peer learning have on trainee clinicians, whether the Aqoon model is effective on a larger scale, and among other healthcare learners, such as those studying nursing or midwifery.

## Data Availability

The datasets generated and analysed during the current study are not publicly available due to the risk that participant privacy could be compromised but anonymised versions can be made available by the corresponding author on reasonable request.

## Abbreviations

AU: Amoud University
HU: Hargeisa University
KCL: King’s College London
KSP: King’s Somaliland Partnership
LMIC: Low and middle income countries
HIC: High income countries
MhGAP: Mental Health Gap Intervention Guide
SGUL: St George University Hospital London
WHO: World Health Organization
